# The Early Diagnosis of Pulmonary Phthisis by the Study of the Mean Temperature

**Published:** 1902-01

**Authors:** J. Tetau


### THE EARLY DIAGNOSIS OF PULMONARY PHTHISIS BY THE STUDY OF THE MEAN TEMPERATURE.*

BY PROF. J. TETAU.

The mean temperature of the human species is 37°, but this temperature may vary or oscillate between 36° and 38°. As body heat is the physical and palpable manifestation of organic reactions, whether chemical or vital, it differs in degree according as the phenomena are more or less acute. The mean temperature of an individual is obtained by taking it in the axilla at 7 A.M. and 5 P.M. for twelve days, under the same conditions of life and habits, and if possible without the patient's knowledge; divide the total sum of the temperatures by the number of times taken and the result will be the mean. The author has made the following classification from his observations of a great number of cases:

1. Those where the internal combustion is too active; the mean temperature of 37.5° and above without passing 38°.

---

*Translated from the *Archives Medicales de Paris*, by Arthur G. Hobbs, M.D., Ophthalmic and Aural Surgeon to the Presbyterian Hospital, Atlanta, Ga.

2. Those nearer normal ranging close to 37°.

3. Those of lessened combustion ranging below 37°, but not beyond 36°.

After long and patient study of the meaning of these varying temperatures, the author has made the following deductions :

1. All individuals whose mean temperature is above 37.5° are predisposed to consumption.

2. All individuals whose mean temperature is 37° to 37.5° are subjects of normal organic changes.

3. All individuals whose mean temperature is below 37° are arthritic, herpitic or scorbutic.

Clinical observations demonstrate that patients with lessening nutrition as a rule have a mean temperature below 37°; the inverse is shown with those whose mean temperature is above 37.5° ; some have too little combustion and others have too much. By applying these laws to a large number of cases Prof. Tetau thinks he has arrived at the bottom cause of the predisposition to pulmonary phthisis.

The numerous observations that he has been able to make upon a stable clientelle, whose family history he has been able to study, have shown him that the predisposition to the receptivity of tuberculosis contagion and the progress of the malady were the direct cause of the elevation of the mean temperature characterizing the organic changes. An individual may become phthisical before he has developed tuberculosis. These two conditions may and do, for the most part, exist together, but can exist independently of each other. This distinction may seem subtle, it is true, but it is of considerable importance from a diagnostic standpoint; one may see pulmonary tuberculosis, not phthisical—that is, presenting only the phenomena of consumption; there exists also phthisis at the beginning which has not yet become tuberculosis, and we have observed, says the author, typical cases of this kind in young people from fourteen to seventeen years of age who had exhibited for five or six months a temperature of 37.8° without any signs of tuberculosis; neither cough, expectoration nor sweats. "Any individual who does not cough has not pulmonary tuberculosis," said Marfau, who ranks with Trouseau as a diagnostician.

In the cases we mentioned above some were cured without evincing any other symptoms than the diminution in the elevation of the mean temperature which declined to the neighborhood of 37° under antiseptic and reconstructive treatment.

Among the numerous persons of all ages and both sexes that have come under his examination, he has remarked that those who contract tuberculosis most easily were those whose mean temperature was above 27.5° This rule is so true that he did not fear to say that four-fifths contract tuberculosis within six months to one year, if not prevented. In those whose temperature was below 27.5°, the predisposition to contract the disease was in proportion as the temperature approached this figure. This easy, though it may be said to be tedious, diagnosis plays no small part in the early therapeutics of the disease.

To epitomize the remainder of this paper, we find the author's conclusions are:

Thus being able early to make an assured diagnosis of phthisis before tubercles have formed, a general antiseptic and constructive treatment can be instituted in time to immune the soil that was ready for the sprouting of any stray bacillus. * * * * *

The translator has seen and treated a number of cases, during the past fifteen years, that were predisposed to consumption by all other means of diagnosis than that of this temperature test, and that, too, may have proven confirmatory, had I resorted to it.

When such cases are complicated with a sub-acute or chronic catarrhal inflammation of the upper respiratory tract, I have rarely failed to restore the patient to a healthy, or at least to an apparently healthy condition, by a daily inhalation for several weeks of a fine oil spray under heavy pressure. This spray has for its base, oil vaseline or liquid alboline, well warmed before being inhaled into the bronchial tubes. It contains in solution such antiseptics and resolvents as are quickly assimilated by the bronchial mucosa, oil terebene, oil eucalyptol, beechwood creosote, etc.

This treatment is on the line of the author's idea: To quickly determine the patient's susceptibility to tuberculosis by the temperature test, and then to render him immune to the growth of stray bacilli that may enter his organism at this most vulnerable point.

These antiseptic inhalations have evinced their efficacy in the frequent cases that I now meet in robust health, who once considered themselves fit subjects for tuberculosis.

